# Mitigating Intestinal Dysbiosis in the Very Preterm Infant

**DOI:** 10.1016/j.advnut.2024.100236

**Published:** 2024-05-20

**Authors:** Mark A Underwood

**Affiliations:** Department of Pediatrics, University of California Davis, Sacramento, CA

The complex intestinal microbiome of the child or adult consists of multiple anatomic niches, each with dozens to hundreds of species and billions to trillions of microbes that remain relatively stable over time due to colonization resistance, i.e., the capacity to return to a baseline community after perturbations such as antibiotics, temporary changes in diet, and probiotics. Prior to the twentieth century, term infants routinely became colonized at birth with microbes from their mother’s birth canal and rectum, which were replaced by obligate anaerobes capable of consuming the many unique glycans in human milk; these relatively few species dominated the infant intestinal microbiome until the introduction of solid foods. With the introduction of antibiotics, cesarean delivery, infant formulas, and modern hygiene practices, the intestinal microbiome of the term infant has changed from that of our ancestors with decreased obligate anaerobes and increased facultative anaerobes. Very preterm infants rarely survived prior to the 1970s and thus represent a new population from an evolutionary aspect with a unique intestinal microbiome that changes over time and is heavily influenced by developmental and environmental factors, with lesser impact from mode of delivery and diet. The “normal” or ideal intestinal microbiome for the very preterm infant remains unclear.

Intestinal dysbiosis implies an alteration in the intestinal microbiome associated with disease. In the child or adult, the classic examples are antibiotic-associated diarrhea and *Clostridium difficile* colitis, although mounting evidence suggests associations between intestinal dysbiosis and a variety of intestinal and systemic diseases with causality demonstrated in many through animal models. In the very preterm infant, the most compelling example of intestinal dysbiosis is necrotizing enterocolitis (NEC), a common and devastating disease with a high-mortality rate. Evidence that intestinal dysbiosis precedes and is a risk factor for NEC in preterm infants includes observational studies of the fecal microbiota in infants with and without NEC [1], increased incidence of NEC with microbiota-altering interventions such as prolonged postnatal administration of antibiotics [2] and acid-blocking agents [3], and decreased incidence of NEC and death with administration of probiotics to preterm infants [4]. The latter has prompted conditional recommendations for routine probiotic prophylaxis for this high-risk group by the WHO, American Gastroenterological Association, and European Society for Paediatric Gastroenterology Hepatology and Nutrition, but not by the American Academy of Pediatrics or the FDA [5].

Mechanisms by which probiotics protect preterm infants include alterations in the composition of the community of microbes (which microbes are present) and in the function of members of this community (what these microbes are consuming and secreting and how they interact with and influence host immune responses including barrier integrity and inflammation). In this issue of *Advances in Nutrition*, He et al. [6] combine and analyze 5 publicly available datasets from studies of probiotic administration to preterm infants across 5 different countries. Their key findings fall into the same broad categories: composition (more commensal bacteria and fewer potential pathogens and proinflammatory bacteria plus maturation of the microbiota as demonstrated by shifts in preterm gut community types) and function (alterations in gene expression by intestinal microbes). Although these findings shed some light on how probiotics impact the developing preterm gut and preterm host-microbe interactions, there is much in each of these 2 broad categories that remains unknown.

## Composition: Alpha Diversity of the Fecal Microbial Community

Alpha diversity refers to diversity within a given sample. In adults and children, low alpha diversity is a marker of dysbiosis, and increased alpha diversity is associated with stability of the microbial community and colonization resistance. In breastfed term infants delivered vaginally and not exposed to antibiotics, low-alpha diversity is common with a relatively few *Bifidobacterium* species dominating the microbiota [7]. He et al. [6] found no impact of probiotic administration to preterm infants on any of the common indices of alpha diversity. The value of alpha diversity in defining dysbiosis in the preterm infant remains unknown.

## Composition: Spatial Diversity of the Intestinal Microbial Community

Although human fecal samples are easily obtained and analyzed, there are profound differences in both host immunology and microbial communities across the various anatomic niches of the gut. How well the fecal microbiome reflects what is happening in the ileum (the most common site for NEC) is uncertain. Addressing this question is challenging as endoscopy entails excessive risk in very preterm infants, and creation of an ostomy quickly and dramatically alters the microbiome. Pairing observations from preclinical models with data from human studies is essential to understanding changes in the microbiome of the preterm ileum.

## Composition: Beta Diversity and Relative Abundance of Key Bacterial Taxa

Beta diversity and assessments of relative abundance are both measures of how 2 or more samples differ from each other. He et al. [6] found that the 2 greatest impacts on beta diversity in preterm infants are age and probiotic administration, with the latter resolving by 36 wk postmenstrual age. Staphylococcaceae and Enterobacteriaceae were the most abundant families and decreased during probiotic administration. Increases in the administered probiotic genera differed based on the probiotic administered (sustained increase in relative abundance during administration of bifidobacteria but not of lactobacilli).

## Function: Competition among Intestinal Microbes

The observation of He et al. [6] that *Bifidobacterium* species outcompeted *Lactobacillus* species when both were administered is consistent with evidence that the nutritional substrate (in this case, human milk oligosaccharides, which are digestible by the former but not the latter) is important to colonization and is confirmed by the significant increase in expression in genes involved in the *Bifidobacterium* shunt in preterm infants receiving probiotics (Supplemental Table 5, pooled *P* value 8.47E-91). Most studies of children and adults show no long-term colonization with probiotic microbes, whereas term infants show stable colonization after a brief administration of a human milk oligosaccharide-consuming probiotic for as long as breast milk is provided [8]. The finding of He et al. [6] that fecal probiotic genera decrease rapidly with cessation of probiotics is consistent with other studies of preterm infants. Secretion of bacteriocins by Gram-positive bacteria and of microcins by Gram-negative bacteria are examples of antibacterial activity directed by microbes against microbes. As bacteriocin production is common among both bifidobacteria and lactobacilli, it may be valuable in future studies to assess the impact of probiotic administration on the expression of bacteriocin-associated genes.

## Function: Competition with the Host

The most compelling example of the host and the intestinal microbes competing for nutrients is iron. The battle for iron in the infant gut lumen between the infant and the gut microbes is influenced by lactoferrin, lipocalin-2, and hepcidin in the former and complex systems to facilitate heme transport and degradation in the latter. In adults, both iron deficiency and iron overload are associated with intestinal dysbiosis. Although small studies suggest minimal impact of lactoferrin administration (reference 28 in He et al. [6]) and iron intake [9] on the fecal microbiota of the preterm infant, studies of the impact of probiotic administration on iron status and associations between iron status and NEC would be valuable.

## Function: Probiotic Metabolites (Postbiotics)

Probiotic microbes produce and secrete a variety of substances that impact surrounding microbes and the host. Perhaps the most compelling of these postbiotic molecules are short-chain fatty acids (predominantly butyrate, propionate, and acetate) and lactic acid. These substances lower the intestinal pH and the luminal oxygen content, creating a less hospitable environment for facultative anaerobes. In addition, butyrate is an important nutrient for host colonocytes, and indole-3-lactate has anti-inflammatory activities [10,11].

Although it is clear that both the composition and the function of the intestinal microbiome are important in the pathogenesis of NEC in preterm infants, there is much that remains unknown. Given that bacterial taxa from different families can perform similar functions, it is likely that measures of microbial function will have more value in understanding NEC and predicting NEC risk than simple measures of composition. Inclusion of functional markers such as fecal pH, redox, and bacterial metabolites such as short-chain fatty acids and lactate in future probiotic studies in preterm infants would add significant value [12].

## Author contributions

The sole author was responsible for all aspects of this manuscript.

## Conflict of interest

MAU has served as chair of the data safety monitoring board for phase 2 and phase 3 multicenter probiotic clinical trials funded by Infant Bacterial Therapeutics; has received travel funds for a conference sponsored by the International Scientific Association for Probiotics and Prebiotics and travel funds and an honorarium for 2 conferences funded by Prolacta Bioscience; and has served as an expert witness in lawsuits involving necrotizing enterocolitis.

## Funding

MAU has received funding for research on the prenatal intestinal microbiota from the NIH (R01HD059127 and R21HD096247). Support for a clinical trial of a probiotic supplement in term infants was received from Evolve Biosystems, currently Infinant Health; this did not include any salary support.
